# Trio-Based Whole-Exome Sequencing Identifies a *De novo EFNB1* Mutation as a Genetic Cause in Female Infant With Brain Anomaly and Developmental Delay

**DOI:** 10.3389/fped.2020.00461

**Published:** 2020-09-01

**Authors:** Ji Yoon Han, Hyun Jeong Kim, Ja Hyun Jang, In Goo Lee, Joonhong Park

**Affiliations:** ^1^Department of Pediatrics, College of Medicine, Catholic University of Korea, Seoul, South Korea; ^2^Department of Radiology, College of Medicine, The Catholic University of Korea, Seoul, South Korea; ^3^Department of Laboratory Medicine and Genetics, Samsung Medical Center, Sungkyunkwan University School of Medicine, Seoul, South Korea; ^4^Department of Laboratory Medicine, College of Medicine, Catholic University of Korea, Seoul, South Korea; ^5^Department of Laboratory Medicine, Jeonbuk National University Medical School and Hospital, Jeonju, South Korea

**Keywords:** trio exome sequencing, *EFNB1* mutation, schizencephaly, global developmental delay, craniofrontonasal dysplasia

## Abstract

**Background:** Craniofrontonasal syndrome is a rare, X-linked disorder in which heterozygous females ironically reported the majority of patients and is caused by in the *EFNB1* gene located at chromosome Xq13.1. Unlike previous reports, we present a female infant with a *de novo EFNB1* missense mutation that was demonstrated in clinical diagnosis as global developmental delay (GDD) and brain anomaly without frontonasal dysplasia or other malformation.

**Case Presentation:** This study reports the genetic analysis of a 4-month-old female infant presenting brain anomaly and GDD. She was the only child of unrelated parents. Early developmental was characterized by delays in fine motor, achieving gross motor, language, and social–cognitive milestones. She could not control her head or hold objects until 4 months of age. Brain magnetic resonance imaging revealed schizencephaly and dysgenesis of corpus callosum. Trio-based whole-exome sequencing revealed a heterozygous c.943C>T (p.Pro315Ser) in the *EFNB1*. Sanger sequencing confirmed this heterozygous alteration occurring in a dominant *de novo* manner, as a consequence of phenotypic and genotypic wild type in both parents.

**Conclusion:** *EFNB1* mutation is considered for a child with schizencephaly, and further study focusing on phenotyping is required to understand the possible contribution of environmental impact and genetic modifier in the expression of *EFNB1*.

## Introduction

Craniosynostoses are malformations of the developing skull in which one or more of the cranial sutures of the skull bone fuse prematurely, thereby affecting brain development and skull shape (1). They have marked allelic and phenotypic heterogeneity and are classified into syndromic and nonsyndromic forms. Syndromic craniosynostoses have a typically monogenic etiology and are related with other malformations. Nonsyndromic craniosynostosis is an isolated finding and is categorized according to the suture(s) involved (2). Indeed, pathogenic variant is well described in six major genes such as *EFNB1, ERF, FGFR2, FGFR3, TCF12*, and *TWIST1* causing the recurrent craniosynostosis syndromes (3): Apert, craniofrontonasal (CFN), Crouzon and Crouzon with acanthosis nigricans, Muenke, Pfeiffer, and Saethre-Chotzen syndrome. Among them, CFN syndrome (CFNS) (OMIM #304110) is an X-linked dominant disease caused by loss-of-function mutations in the ephrin B1 gene (*EFNB1*, OMIM ^*^300035). The ephrin-Eph signaling pathway affects the cellular cytoskeleton, leading to cell repulsion primarily as well as to cell adhesion in some instances. This pathway plays a critical role in morphogenesis activating signaling pathways and allowing cell-to-cell communication over a short distance (4). Classically, CFNS is characterized by severe hypertelorism with a central nasal groove related to unilateral coronal craniosynostosis, other midline defects, dermatological manifestations, body asymmetry, and skeletal abnormalities (5). Most CFNS patients are females as heterozygous with respect to X-linked gene who show paradoxically a more severe symptom than hemizygous males, who have an uncertain clinical manifestation (6).

The current report demonstrates a female infant with CFNS caused by a previously unreported *EFNB1* mutation that demonstrates the difference in clinical features including global developmental delay (GDD) and brain anomaly without frontonasal dysplasia or other malformations.

## Case Presentation

**Figure 1:**
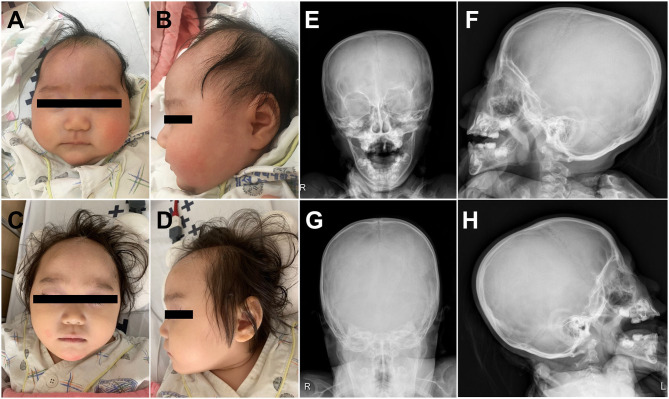
Clinical photographs of the proband showed microcephaly without craniofrontonasal dysmorphism at age of 4 months **(A,B)** and at age of 2 years **(C,D)**. Skull X-ray of the proband revealed no craniosynostosis **(E–H)**.

A 4-month-old female infant was referred to the Department of Pediatric Neurology of Daejeon St. Mary's Hospital with a diagnosis of microcephaly and developmental delay. There was no family history of neurodevelopmental or genetic disorders, and she was the only child of Korean nonconsanguineous parents. The pregnancy had been uneventful. At age 4 months, the weight was 6.6 kg (25th percentile), length was 63 cm (50th percentile), and head circumstance was 35.5 cm (less than the third percentile). Her early development was characterized by delays in fine motor, achieving gross motor, language, and social–cognitive milestones. She could not control her head or hold objects until 4 months of age. Estimation using the Bayley Scale of Infant and Toddler Development, Third Edition, was performed at age 2 years and demonstrated marked GDDs (language cognitive, developmental, and motor ages: 8, 13, and 9–10 months, respectively). At age of 2 years, she was in the 20th percentile for weight (9.6 kg), the 50th percentile for height (81 cm), and first percentile for head circumference (43 cm). Physical examination demonstrated a well-nourished girl with microcephaly at age of 2 years and without CFN dysplasia (Figures 1A–D). Estimated orbital parameters at age of 2 years were all within the 10th percentile to 25th percentile (interpupillary distance 43 mm, inner canthal distance 24 mm, and outer canthal distance 71 mm). The palpebral fissures slightly sloped upward. Skull X-ray revealed no gross abnormality in cranial vault and no craniosynostosis (Figures 1E–H). Her ears, feet, and hands were normal in shape and size (Supplementary Figure 1). Skeletal surveillance including spine, both hands, and foot was done, and results were normal (Supplementary Figure 1). Deep tendon reflexes, muscle bulk, and muscle tone were normal. Brain magnetic resonance imaging revealed schizencephaly and dysgenesis of corpus callosum (Supplementary Figure 2). Her audiometry test result (auditory evoked potential and auditory brain stem response) was normal, and her response to sound seems to appropriate. The results of visual evoked potential and ophthalmologic tests were normal, but her visual perception was slow and did not seem accurate. Ophthalmological examinations of fundus and optic nerve were normal at ages 6 months and 2 years. Electroencephalography (EEG) observed continuous high-amplitude spikes and slow waves on both temporoparietal areas (Supplementary Figure 2). Despite abnormalities in EEG, she did not have a history of seizures. Pelvic sonogram screen reported no malformations. Echocardiogram showed no structural abnormalities. The laboratory tests such as growth hormone, thyroid function test, and metabolic workup were all within normal ranges.

## Trio-Based Whole Exome Sequencing

The study protocol was approved by the Institutional Review Board of the Catholic University of Korea. Written informed consent was collected from the parents on behalf of their children for the publication of any potentially identifiable images or data included in this article before blood sampling, and clinical data were achieved from the proband and her parents. The exomic DNA of the proband and her parents was enriched using the Agilent's SureSelect XT Human All Exon v5 (Agilent Technologies, Santa Clara, CA, USA), and paired-end sequencing was conducted on the Illumina HiSeq2500 (Illumina, San Diego, CA, USA). As a result, rare inherited or sporadic variants in each trio were identified by the mirTrios program based on the VCF files generated by GATK. Annotation of the identified variants with respect to the results of their mutation on reported genes (amino acid change, functional effect, etc.), mutation effect predicted by several *in silico* computational tools (MutationTaster, Polyphen2, SIFT, CADD-PHRED, etc.), and public genome databases (from 1,000 Genomes, ExAC, and gnomAD database) were estimated using the dbNSFP 2.4 and Variant Effect Predictor, which is a database constructed for annotation of presumptive nonsynonymous variants and functional prediction in the human genome.

## Results

**Figure 2:**
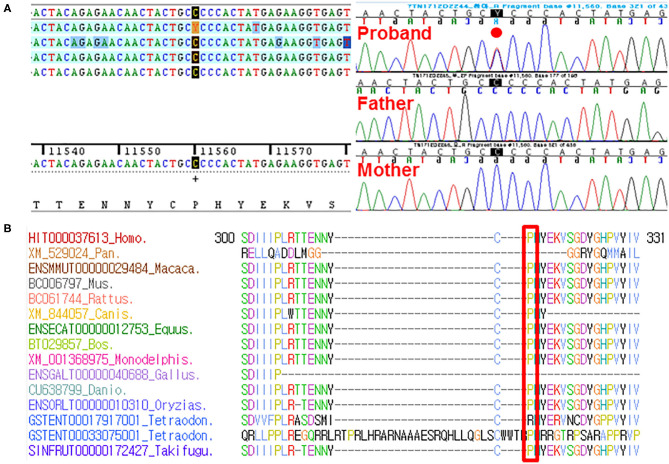
**(A)** Sanger sequencing confirmed this heterozygous c.943C>T (p.Pro315Ser) in the *EFNB1* occurring in a dominant *de novo* manner, as a consequence of phenotypic and genotypic wild type in both parents. **(B)** Sequence alignment of the conserved cytoplasmic domain of the EFNB1 protein in multiple species. Protein sequence of the Pro315Ser residue is highly conserved across species compared. It is highlighted in the red box.

By estimating sequence quality along all sequences, average yield in target of 5,411 million reads was generated from the proband and her parents' samples. Mean depth (*x*) was 107, and percentage of bases above average 30*x* was achieved for the average target region of 91%. Trio-based whole-exome sequencing (WES) identified 21 rare variants as a candidate cause of schizencephaly and GDD (Table 1). Among them, only two rare variants of the *EFNB1* and *ABCC4* were inherited as a dominant *de novo* manner. Although two candidate missense variants were identified, there was no phenotypic description related to brain anomaly and developmental delay in OMIM database due to *ABCC4* variant. Therefore, the *ABCC4* variant seems to be less likely to be the pathogenic variant. Particularly, similar to the phenotype of the proband, a heterozygous c.943C>T of the *EFNB1* gene causing a codon change of proline to serine at position 315 (NM_004429.4: c.943C>T, p.Pro315Ser; no rsID) had not been previously reported to be related to CFNS in the proband. Sanger sequencing was conducted in the proband and her parents to confirm that the mutation segregated with affected individuals and demonstrated this mutation as a dominant *de novo* state was present only in the proband but not in her parents showing phenotypic and genotypic wild type, respectively (Figure 2A). Allele frequency at this position has neither been reported in the gnomAD exome data or in 622 ethnically matched Korean, unrelated controls (http://coda.nih.go.kr/coda/KRGDB/index.jsp). This missense variant of the *EFNB1* was predicted to be “deleterious,” “damaging,” or “disease causing” by computational *in silico* prediction (Supplementary Table 1). In addition, cross-species comparisons (phastCons and GERP) of protein sequences of *EFNB1* protein demonstrated that this mutated region was conserved highly in vertebrates (phastCons 1 > cutoff of 0.8 and GERP 5.04 > cutoff of 4.4). An *EFNB1* multiple sequence alignment of amino acids 300–331 was constructed using the following taxa showing phylogenetic diversity between fugu (Takifugu: SINFRUT00000172427) and human (*Homo sapiens*: HIT000037613) and using Evola (Figure 2B). As a result, amino acid sequence of the Pro315Ser residue is highly conserved across the species.

**Table 1:** Summary of 21 rare variants as a candidate cause of schizencephaly and global developmental delay identified by trio-based exome sequencing.

**Gene**	**Nucleotide ID**	**Base change**	**Codon change**	**Effect**	**rsID**	**Phenotype OMIM**	**Inheritance**
*EFNB1*	NM_004429.4	c.943C>T	p.Pro315Ser	Missense	na	# 304110	*De novo*
*WDR64*	NM_144625.4	c.1568C>G	p.Thr523Arg	Missense	na	na	Maternal
*COL6A3*	NM_004369.3	c.6772G>A	p.Gly2258Ser	Missense	rs1489977815	# 158810, # 616411, #254090	Maternal
*CLDN1*	NM_021101.4	c.163G>A	p.Val55Met	Missense	na	# 607626	Paternal
*IL17RB*	NM_018725.3	c.515dupA	p.Cys173Valfs*16	Frameshift	rs572836124	na	Paternal
*BMP6*	NM_001718.4	c.353_355dup	p.Gln118dup	Frameshift	rs201486498	na	Paternal
*OXR1*	NM_001198532.1	c.764G>A	p.Gly255Asp	Missense	na	na	Paternal
*SHARPIN*	NM_030974.3	c.872A>G	p.Asp291Gly	Missense	rs1378764618	na	Maternal
*SLC20A2*	NM_001257180.1	c.1333_1344del	p.Ile445_Glu448del	Frameshift	rs1316718967	# 213600	Paternal
*B4GALT1*	NM_001497.3	c.533T>G	p.Val178Gly	Missense	na	# 607091	Paternal
*ACSM6*	NM_207321.2	c.917T>C	p.Leu306Pro	Missense	rs1375686523	na	Maternal
*FRMD4A*	NM_018027.3	c.2663_2665dup	p.Gly888dup	Frameshift	rs536647518	# 616819	Maternal
*ADAMTS15*	NM_139055.2	c.361G>T	p.Gly121Trp	Missense	rs1400093741	na	Maternal
*RNF169*	NM_001098638.1	c.1235G>A	p.Arg412His	Missense	rs1309338384	na	Maternal
*TMEM106C*	NM_001143842.1	c.170C>T	p.Thr57Ile	Missense	na	na	Paternal
*ABCC4*	NM_005845.4	c.3217A>T	p.Ile1073Phe	Missense	na	na	*De novo*
*GAN*	NM_022041.3	c.584T>C	p.Val195Ala	Missense	na	# 256850	Paternal
*NOL4*	NM_003787.4	c.1387delC	p.Leu463Serfs*7	Frameshift	na	na	Maternal
*ZNF444*	NM_018337.3	c.511_513del	p.Ala171del	Frameshift	na	na	Paternal

*na, not available*.

## Discussion

Craniosynostosis comprises a heterogeneous group of disorders correspondingly, in addition to monogenic causes, with reduced transduction of stretch forces from the growing brain due to poor intrinsic growth, intrauterine fetal head constraint, and polygenic background all likely to play substantial roles (7). Occasionally, extracranial manifestations include asymmetry of the pectoralis, breast, limbs, or thoracic skeleton; clinodactyly of the fifth finger, polydactylism, and diaphragmatic hernia are seen (8–12). Developmental delay or intellectual disability is not usually present (13–15). Our case showed severe GDD with schizencephaly and microcephaly, but no other skeletal anomaly or facial dysmorphism. However, mild to moderate intellectual disability or developmental delay has been reported in some patients, and it is somewhat related to uncorrected craniosynostosis. Neurocognitive development in CFNS is usually reported as normal in most published cases (16–18). It is not clear whether GDD represents primary features of CFNS or is secondary to the influence of premature fusion of sutures. Our patient had minor facial dysmorphisms such as hypertelorism, slightly broad nasal root, and palpebral fissures slightly sloped upward. However, compared to previous reports, our patient did not show definite facial dysmorphism and craniosynostosis. However, our patient shows severely retarded development at age of 2 years and schizencephaly, which is described as a new manifestation for the first time. In our case, trio-based WES results showed a heterozygous c.943C>T (p.Pro315Ser) in the *EFNB1*. This variant corresponds to the term “likely pathogenic” according to the American College of Medical Genetics guidelines: PS2, *de novo* in a patient with the disease and no family history; PS4, the prevalence of the variant in affected individuals is significantly increased compared with the prevalence in controls; PM5, novel missense change at an amino acid residue where a different missense change determined to be pathogenic has been seen before. In previous study, prior to referral in the majority of cases, the number of *EFNB1* variants was identified in individuals who had not been diagnosed clinically with CFNS (19). This finding supports that *EFNB1* has a broader phenotypic spectrum, and a wider role in undiagnosed craniosynostosis, than previously recognized. The inquiry of whether mutations in other ephrins, in their receptors, or in membrane of the signaling cascade may lead to brain malformations and CFN dysplasia needs to be researched.

**Figure 3:**
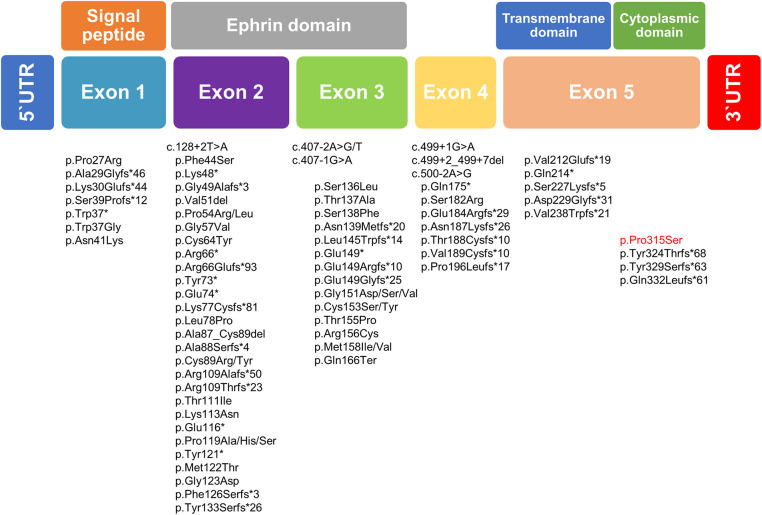
Schematic diagram of the *EFNB1* mutation spectrum in patients with CFNS. Below the putative EFNB1 protein structure mutation identified in the present study is highlighted in red, whereas those published previously are written in black.

The mutation classifications in 89 cases included 3 whole-gene deletions, 3 intragenic deletions, 24 frameshifts, 13 nonsense mutations, 40 missense mutations, and 6 splice site mutations (5, 20–26). *EFNB1* mutations have been dispersed across the gene, but mutations in exon 5, as found in our family, may be distributed infrequently compared with mutations in the first four exons of *EFNB1*. The most mutations were identified in exons 2 and 3 of *EFNB1* gene encoding the extracellular ephrin domains (21). In our study, missense mutation c.943C>T was identified and results in the change of p.Pro315Ser located in exon 5 of *EFNB1* gene coding the cytoplasmic domain (Figure 3). To date, most of the mutations in exon 5 have been deleterious mutations such as frameshift or nonsense mutations interfering with ephrin B1 reverse signaling that contribute to the CFNS phenotype. (19, 21, 23, 27). Unlike previous reports, this is the report missense *EFNB1* mutation in exon 5 in atypical CFNS with brain anomaly but no CFN dysplasia. The effects of a missense mutation on molecular phenotype, function, and organism fitness can be extremely diverse. Alternatively, it may be mildly deleterious to compare splice site, frameshift, or nonsense mutations that can be interpreted easily as pathogenic in nature (28). Thus, the *EFNB1* mutation type may have conferred a different phenotype in our case from those in previous studies. In addition, genotype and phenotype difference between ephrin (*n* = 62) and cytoplasmic (*n* = 7) domains was estimated; however, because of the small number of patients with mutation in cytoplasmic domain, it is difficult to fully verify the genotype–phenotype correlation (Supplementary Table 2).

High heterogeneity due to overlapping clinical features, as well as differences in expressivity and penetrance observed in craniosynostosis cases, often makes correct clinical diagnosis extremely difficult (2). Molecular genetic analysis is necessary to link as many phenotypes as possible with their underlying genetic cause, building the base for proper genotype–phenotype correlations, allowing for prediction of patient's clinical course and exact genetic counseling (14, 19, 29). The high apparent rate of rare actionable mutations detected in complex craniosynostosis without an obvious diagnosis may reflect developmental pleiotropy of signaling in the cranial sutures, with co-opting from more ancient uses in embryogenesis and multiple pathways, implicated at different stages of suture development (7, 19, 30, 31). As a result of the genetic diagnosis, potentially appropriate monitoring has been instigated.

In summary, *EFNB1* mutation is considered for a child with schizencephaly, and further study focusing on phenotyping is required to understand the possible contribution of environmental impact and genetic modifier in the expression of *EFNB1*. Our case with atypical phenotype showed uncommon features. Most mutations were located in the ephrin domain (exons 2 and 3), and only some patients have mutation in cytoplasmic domain (exon 5). Mutation in the cytoplasmic domain (exon 5) may result in lesser extracranial abnormalities compared to mutation in the ephrin domain. Still, further analysis with more patients is necessary in order to establish the genotype–phenotype relations.

## Data Availability Statement

The raw data supporting the conclusions of this article will be made available by the authors, without undue reservation.

## Ethics Statement

The study protocol was approved by the Institutional Review Board of the Catholic University of Korea. Written informed consent to participate in this study was provided by the participants' legal guardian/next of kin. Written informed consent was obtained from the individual(s), and minor(s)' legal guardian/next of kin, for the publication of any potentially identifiable images or data included in this article.

## Author Contributions

JH made substantial contributions to interpretations of clinical medical record and was involved in drafting the manuscript. HK made substantial contributions to analysis radiologic findings. JJ contributed to the acquisition and interpretation of NGS data. JP made substantial contribution to analysis and interpretation of the data and was involved in drafting manuscript. IL was involved in revising critically for important intellectual content. All authors read and approved the manuscript for submission, contributed to the article, and approved the submitted version.

## Conflict of Interest

The authors declare that the research was conducted in the absence of any commercial or financial relationships that could be construed as a potential conflict of interest.

## Supplementary Material

Supplementary Figure 1 X-ray of the proband shows no gross abnormality in both feet **(A, B)** and both hands **(C, D)**, and whole spine **(E,F)**.

Supplementary Figure 2 Brain MRI performed at 2 years of age in the patient. T2-weighted axial **(A)** and coronal **(B)** MR images demonstrate bilateral CSF-filled brain defects extending from the lateral ventricle wall to pial surface and dysplastic gray matter lining the clefts (red arrows). **(C)** T1-weighted sagittal MR image shows focally thinned and dysgenetic corpus callosum (red arrow). **(D)** EEG shows continuous spikes and slow waves on both temporooccipital areas.

Supplementary Table 1 Results of predictive *in-silico* tools on missense variants identified by trio-based exome sequencing.

Supplementary Table 2 Comparison of clinical abnormalities according to *EFNB1* mutation sites (5, 20–26).
