# Supplementary figures and images for: Trio-Based Whole-Exome Sequencing Identifies a De novo EFNB1 Mutation as a Genetic Cause in Female Infant With Brain Anomaly and Developmental Delay
Source: Front Pediatr. 2020 Sep 1;8:461. doi: 10.3389/fped.2020.00461 (PMC7490291; doi:10.3389/fped.2020.00461)

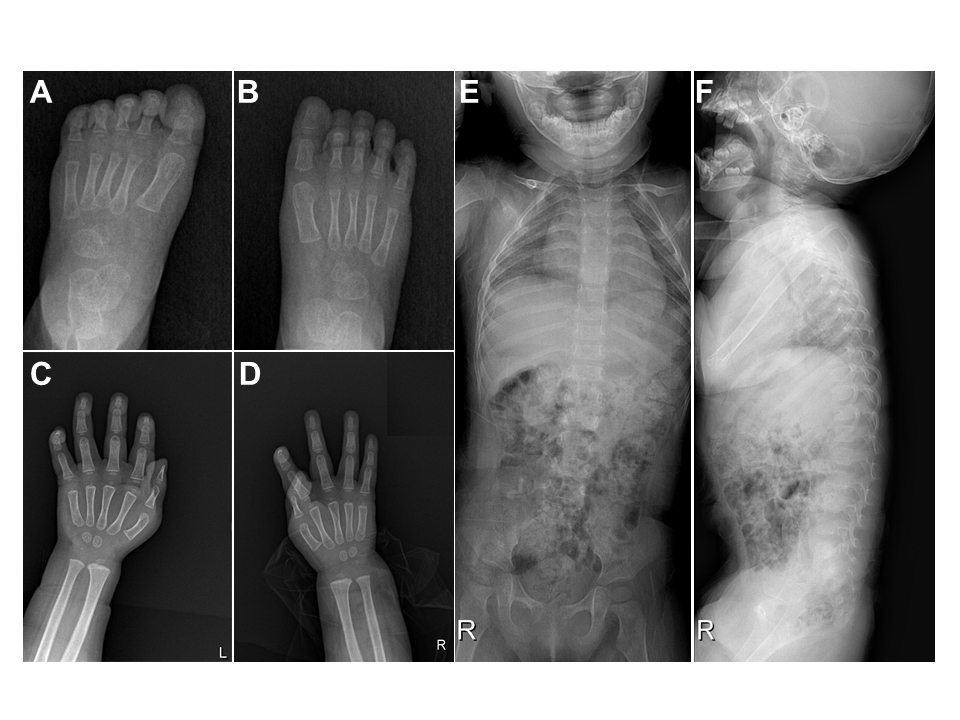

Supplement: Supplementary Figure 1 — X-ray of the proband shows no gross abnormality in both feet (A, B) and both hands (C, D), and whole spine (E,F). [file Image_1.TIF]

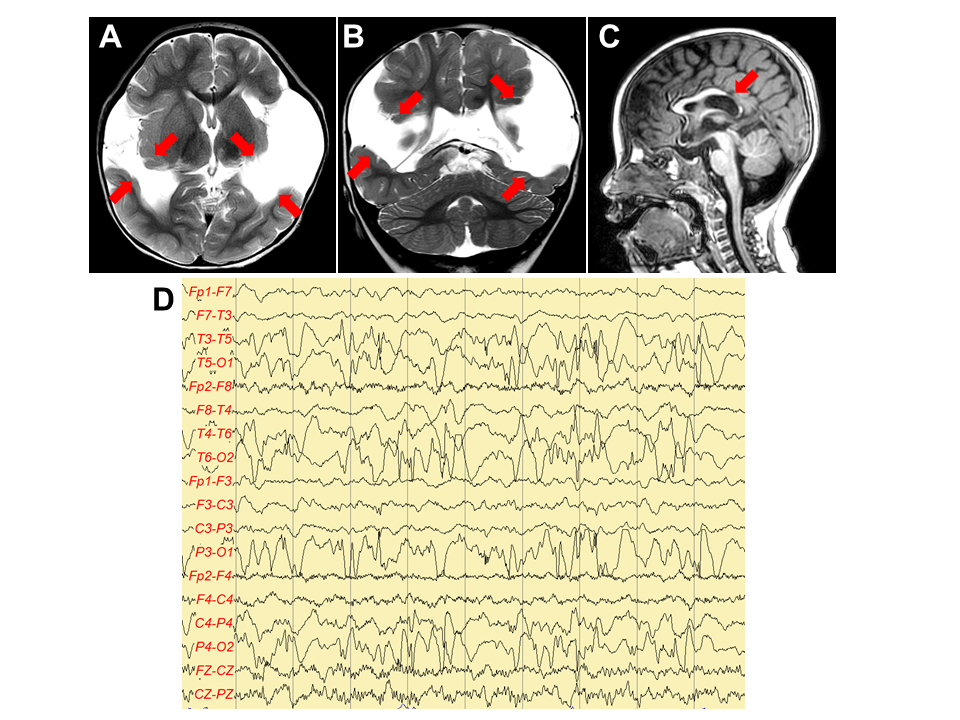

Supplement: Supplementary Figure 2 — Brain MRI performed at 2 years of age in the patient. T2-weighted axial (A) and coronal (B) MR images demonstrate bilateral CSF-filled brain defects extending from the lateral ventricle wall to pial surface and dysplastic gray matter lining the clefts (red arrows). (C) T1-weighted sagittal MR image shows focally thinned and dysgenetic corpus callosum (red arrow). (D) EEG shows continuous spikes and slow waves on both temporooccipital areas. [file Image_2.TIF]
