# Supplementary material for: Trio-Based Whole-Exome Sequencing Identifies a De novo EFNB1 Mutation as a Genetic Cause in Female Infant With Brain Anomaly and Developmental Delay
Source: Front Pediatr. 2020 Sep 1;8:461. doi: 10.3389/fped.2020.00461 (PMC7490291; doi:10.3389/fped.2020.00461)
Supplement: Supplementary Table 1 — Results of predictive in-silico tools on missense variants identified by trio-based exome sequencing. [file Table_1.docx]

| **Supplementary Table S1. Results of predictive in-silico tools on missense variants identified by trio-based exome sequencing** | | | | | | | | |
| --- | --- | --- | --- | --- | --- | --- | --- | --- |
| **Gene** | **SIFT** | **Polyphen2** | **LRT** | **MutationTaster** | **CADD- PHRED** | **GERP** | **phastCons100way** | **gnomAD (exome freq.)** |
| *EFNB1* | Deleterious | Damaging | Deleterious | Disease-causing | 26.4 | 5.04 | 1 | na |
| *WDR64* | Deleterious | Damaging | Deleterious | Disease-causing | 28.0 | 6.06 | 0.996 | na |
| *COL6A3* | Deleterious | Damaging | Deleterious | Disease-causing | 27.1 | 5.42 | 0.999 | 0.000007953 |
| *CLDN1* | Deleterious | Damaging | Deleterious | Disease-causing | 32 | 5.96 | 1 | na |
| *OXR1* | Deleterious | Damaging | Deleterious | Disease-causing | 27.4 | 5.27 | 1 | na |
| *SHARPIN* | Deleterious | Damaging | Deleterious | Disease-causing | 27.4 | 4.63 | 0.988 | 0.000004012 |
| *B4GALT1* | Deleterious | Damaging | Deleterious | Disease-causing | 28.9 | 5.18 | 1 | na |
| *ACSM6* | Deleterious | Damaging | na | Disease-causing | 24.3 | 1.21 | 0.994 | 0.000007959 |
| *ADAMTS15* | Deleterious | Damaging | na | Disease-causing | 29.7 | 4.63 | 1 | 0.000004113 |
| *RNF169* | Deleterious | Damaging | Deleterious | Disease-causing | 28.4 | 5.99 | 1 | 0.000008016 |
| *TMEM106C* | Deleterious | Damaging | Deleterious | Disease-causing | 28.9 | 4.24 | 1 | na |
| *ABCC4* | Deleterious | Damaging | Deleterious | Disease-causing | 27.8 | 6.14 | 1 | na |
| *GAN* | Deleterious | Damaging | Deleterious | Disease-causing | 24.8 | 5.8 | 1 | na |
| na, not available | | | | | | | | |
