# Supplementary material for: Trio-Based Whole-Exome Sequencing Identifies a De novo EFNB1 Mutation as a Genetic Cause in Female Infant With Brain Anomaly and Developmental Delay
Source: Front Pediatr. 2020 Sep 1;8:461. doi: 10.3389/fped.2020.00461 (PMC7490291; doi:10.3389/fped.2020.00461)
Supplement: Supplementary Table 2 — Comparison of clinical abnormalities according to EFNB1 mutation sites (5, 20–26). [file Table_2.docx]

| **Supplementary Table S2. Comparison of clinical abnormalities according to *EFNB1* mutation sites^5, 20-26^** | | |
| --- | --- | --- |
| **Clinical abnormalities** | **Ephrin domain** | **Cytoplasmic domain** |
| Head  Craniosynostosis or brachycephaly | 52/62 (84%) | 6/7 (86%) |
| Brain anomaly | 11/62 (18%) | 2/7 (29%) |
| Facial dysmorphism | 61/62 (98%) | 6/7 (86%) |
| Thorax | 6/62 (10%) | 0/7 (0%) |
| Limbs | 13/62 (21%) | 1/7 (14%) |
| Genitalia | 3/62 (5%) | 0/7 (0%) |
| Neurodevelopmental problems | 9/62 (15%) | 1/7 (14%) |
| Others | 11/62 (18%) | 1/7 (14%) |
